# Advances in understanding the role of inflammatory factors and immune cells in the pathology of epilepsy, mediated by neuroimmune interactions within the gut-brain axis

**DOI:** 10.3389/fcell.2025.1650909

**Published:** 2025-11-11

**Authors:** Lijia Zhang, Rongjiang Xu, Hao Huang, Juan Yang, Changyin Yu, Haiqing Zhang, Zucai Xu

**Affiliations:** 1 Department of Neurology, Affiliated Hospital of Zunyi Medical University, Zunyi, China; 2 Key Laboratory of Brain Function and Brain Disease Prevention and Treatment of Guizhou Province, Zunyi, China

**Keywords:** epilepsy, inflammation, gut-brain axis, neuron, inflammatory factors

## Abstract

Epilepsy is a prevalent chronic neurological disorder, affecting approximately 70 million individuals globally, with its pathogenesis primarily attributed to recurrent seizures caused by abnormal neuronal discharges in the brain. Recent research has increasingly recognized the critical role of neuroinflammation in the central nervous system in the onset and progression of epilepsy. Furthermore, the gut-brain axis, a crucial link between gut microbiota and the central nervous system, facilitates communication through intricate pathways involving neural, immune, and endocrine mechanisms, and its involvement in epilepsy pathology is gaining significant attention. This review focuses on recent advances in neuroimmune interactions within the gut-brain axis in epilepsy. It explores the roles of inflammatory factors (e.g., IL-1β, IL-6, TNF-α) and immune cells (e.g., microglia, macrophages, neutrophils) in epileptic pathophysiology, and systematically reviews relevant experimental and clinical studies. The article begins by providing an overview of the fundamental interactions between gut microbiota and the host immune system, before discussing how gut-derived immune signals influence the central nervous system via the gut-brain axis. The pathogenic mechanisms of key pro-inflammatory factors in epileptogenesis are then examined, including how IL-1β promotes neuronal hyperexcitability, how IL-6 mediates neuroinflammation, and how TNF-α disrupts the balance between neuronal excitation and inhibition. Additionally, the article highlights the significant role of inflammatory cells in the central nervous system, particularly the activation of microglia and the infiltration of peripheral immune cells in epilepsy development. In conclusion, further investigation into the mechanisms of neuroimmune interactions in the gut-brain axis may lead to the identification of novel biomarkers and therapeutic targets for epileptogenesis, offering new insights and directions for the treatment of refractory epilepsy.

## 1 Introduction

Epilepsy is the third most prevalent chronic neurological disorder, following stroke and dementia, and is characterized by recurrent seizures due to abnormal hypersynchronous neuronal discharges in the brain (Scheffer et al., 2024; Nabbout et al., 2023). Globally, over 70 million individuals are affected by epilepsy, with a bimodal prevalence distribution observed in pediatric and elderly populations (GBD 2016 Epilepsy Collaborators, 2019a; GBD 2016 Neurology Collaborators, 2019b). This disorder significantly impacts patients’ quality of life and imposes a substantial socioeconomic burden. Despite recent advancements in antiepileptic drugs and surgical treatments, approximately 30% of epilepsy patients remain resistant to available therapies and fail to achieve satisfactory seizure control (Pong et al., 2023). The pathogenesis of epilepsy is multifactorial, involving abnormalities in ion channel function, an imbalance between excitatory and inhibitory neurotransmitters, and genetic factors (Oliver et al., 2023; Ng et al., 2024). In recent years, neuroinflammation has been increasingly recognized as a key mechanism in epileptogenesis and the formation of epileptic foci (Liu et al., 2023; Guo et al., 2022). Numerous studies have shown that epileptic seizures can initiate an inflammatory response in the central nervous system (CNS), and chronic, persistent inflammation can lower the brain’s convulsive threshold, thus promoting epilepsy and create a vicious cycle of “inflammation-epilepsy.” For instance, various pro-inflammatory mediators, including interleukin-1β (IL-1β), IL-6, high mobility group protein 1 (HMGB1), and tumor necrosis factor-alpha (TNF-α), have been found to be elevated in both epilepsy patients and animal models (Soltani Khaboushan et al., 2022; Williams et al., 2022; Chen et al., 2025). Additionally, inhibiting certain inflammatory pathways has demonstrated antiepileptic effects in animal studies (Qin et al., 2022; Hanin et al., 2023).

It is important to note that the central nervous system is not an isolated “immune zone”; instead, it interacts with the peripheral immune system through multiple pathways (Yue et al., 2022; Amanollahi et al., 2023). The gut-brain axis, in particular, serves as a critical link between gut microbiota and CNS activity, involving neural pathways (such as the vagus nerve and enteric nervous system), endocrine pathways (such as the hypothalamic-pituitary-adrenal axis), and immune/metabolic pathways (Mazarati, 2024). The human gut, rich in microbial communities and immune tissues, is considered the body’s largest immune organ. Gut microbes interact with the intestinal mucosal immune system via their metabolites and mycobacterial components, influencing systemic immune homeostasis (Li et al., 2025; Riva et al., 2024b). In turn, alterations in immune system function can affect brain activity through humoral, cytokine, and vagal pathways. Studies have indicated that intestinal dysbiosis may activate microglia and trigger inflammatory cascades within the CNS, subsequently affecting seizure susceptibility (Mula et al., 2022). Furthermore, seizures and prolonged medication use can alter the composition of the gut microbiota, as evidenced by significant alterations in the diversity and abundance of gut flora in epilepsy patients (Perucca et al., 2023).

In conclusion, neuroimmune interactions within the gut-brain axis have emerged as a promising area of epilepsy research. This review will focus on the mechanisms and evidence underpinning the interaction between gut microbiota and the host immune system in the development of epilepsy. It will highlight the roles of pro-inflammatory factors and immune cells in this process and explore potential therapeutic strategies targeting the gut microbiota or immune system. Finally, the review will address the challenges and future research directions in this field. In particular, this work emphasizes the critical role of the gut-brain axis in linking neuroinflammation to epileptogenesis.

## 2 Mechanisms of inflammatory factors in epilepsy

**FIGURE 1:**
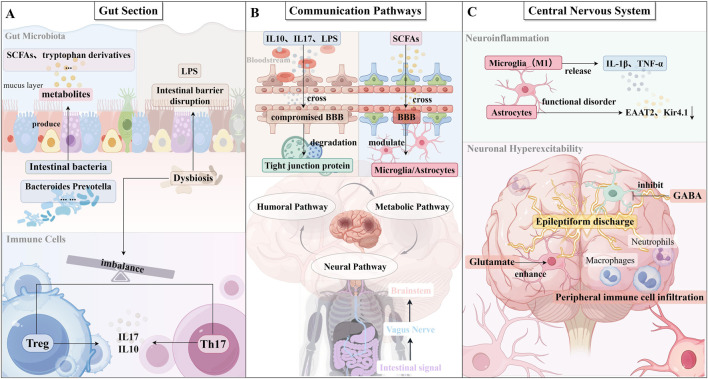
**(A)** Gut compartment: Gut microbiota dysbiosis characterized by altered abundance of Firmicutes and Bacteroidetes phyla, leading to abnormal production of microbial metabolites (short-chain fatty acids [SCFAs] and tryptophan derivatives). Immune dysregulation with Th17/Treg imbalance results in elevated IL-17 and reduced IL-10 levels. Disrupted intestinal barrier integrity increases permeability, allowing bacterial lipopolysaccharide (LPS) translocation into systemic circulation. **(B)** Communication pathways: Humoral pathway: Pro-inflammatory mediators (IL-1β, TNF-α) and LPS cross the compromised blood-brain barrier (BBB) through degraded tight junction proteins (e.g., claudin-5, occludin). Neural pathway: Gut-derived signals are transmitted via the afferent vagus nerve to the nucleus tractus solitarius (NTS) in the brainstem. Metabolic pathway: SCFAs (e.g., butyrate) cross the BBB and modulate microglia and astrocyte function. **(C)** Central nervous system effects:Neuroinflammation: Activated M1 microglia release pro-inflammatory cytokines (IL-1β, TNF-α). Astrocytes exhibit dysfunctional glutamate clearance (↓EAAT2) and potassium buffering (↓Kir4.1). Neuronal hyperexcitability: Imbalanced excitatory (↑glutamate) and inhibitory (↓GABA) neurotransmission. Infiltrating peripheral immune cells (macrophages, neutrophils) exacerbate neuroinflammation. (By Figdraw).

A substantial body of research has elucidated the role of pro-inflammatory cytokines in the onset and persistence of epilepsy (Lee et al., 2020a). These inflammatory factors, primarily produced by activated central glial cells (microglia and astrocytes) and infiltrating peripheral immune cells, influence neuronal excitability and synaptic plasticity through multiple pathways, thereby lowering seizure thresholds and potentially contributing to epileptogenesis (Hermann et al., 2017; Ding et al., 2022). This section highlights the mechanisms through which several inflammatory mediators, most closely associated with epileptogenesis (Figure 1), including IL-1β, IL-6, IL-17, TNF-α, and high mobility group proteins such as HMGB1, affect epileptic pathophysiological processes, reviewing relevant experimental evidence (Zhang et al., 2024a; Jiang et al., 2023; Lee et al., 2020b). A consolidated overview of these mediators and their principal cellular interactions is summarized in Table 1.

**TABLE 1:** Interactions between major inflammatory mediators and immune cells in epilepsy.

Mediator	Primary source cells	Target/Interacting cells	Main effects	Contribution to epilepsy
IL-1β	Microglia, astrocytes, damaged neurons	Neurons, astrocytes	↓ GABA_A receptor currents; ↑ glutamate release; ↑ COX-2/PGE2; triggers HMGB1 release	Enhances excitatory drive, reduces inhibition, lowers seizure threshold
IL-6	Astrocytes, microglia (induced by IL-1β, TNF-α, IL-17)	Neurons, progenitor cells	↑ glutaminase, ↑ AMPA receptors; ↓ LTP and neurogenesis; maternal IL-6 affects offspring	Facilitates hyperexcitability, promotes seizures; biphasic role (pro-convulsant vs. neuroprotective in chronic phase)
IL-17	Th17 cells, astrocytes, neurons	Endothelial cells, neurons, glia	↑ chemokines (CCL2) → immune infiltration; ↓ GABAergic inhibition; positive feedback with IL-1β/TNF-α	Breaks BBB, recruits immune cells, amplifies inflammation, correlates with seizure severity
TNF-α	Microglia, astrocytes, macrophages	Neurons, endothelial cells, T cells	↑ glutamate release, ↑ AMPA clustering; ↓ GABA_A receptors; ↑ ICAM-1 → T cell infiltration	Promotes hyperexcitability; biphasic dose effect via TNFR1 (pro-inflammatory) and TNFR2 (neurotrophic/feedback)
HMGB1	Damaged neurons, activated microglia/astrocytes	Microglia, neurons (via TLR4, RAGE)	Acts as DAMP; ↑ IL-1β, TNF-α; ↑ NMDA receptor activity; disrupts BBB tight junctions	Sustains inflammation, neuronal hyperexcitability, and epileptiform discharges

### 2.1 Interleukin-1β (IL-1β)

IL-1β is one of the most extensively studied pro-inflammatory cytokines in the central nervous system. The expression of IL-1β and its receptor, IL-1R1, is significantly upregulated in postoperative focal brain tissues of epilepsy patients and in various animal models of epilepsy (Lee et al., 2020b; Rana and Musto, 2018). IL-1β is predominantly released by activated microglia, astrocytes, and damaged neurons, and it activates the downstream MyD88/NF-κB signaling pathway through the IL-1R1 receptor, inducing the expression of numerous inflammatory genes (Jiang et al., 2023; Lee et al., 2020b; Rana and Musto, 2018). In the context of epilepsy, IL-1β exerts several pro-seizure effects:

Reduction of Inhibitory GABAergic Transmission: Electrophysiological studies have shown that IL-1β exposure reduces the amplitude of GABA_A receptor-mediated currents in hippocampal neurons by approximately 30%, suggesting that IL-1β attenuates postsynaptic inhibitory potentiation. Roseti et al. observed that in tissue slices of human temporal lobe epileptic foci, the addition of IL-1β markedly reduced GABA_A receptor-mediated currents, and the use of an IL-1 receptor antagonist (IL-1Ra) blocked this effect. A reduction in inhibitory transmission disrupts the excitatory/inhibitory balance, increasing the likelihood of overexcitation of the neural network (Horn et al., 2022; Sanz and Garcia-Gimeno, 2020; Kamali et al., 2021).

Enhancement of Excitatory Glutamatergic Transmission: IL-1β promotes the inactivation of the intracellular glutamine synthetase (GS) in astrocytes, impairing the clearance and recycling of synaptic glutamate and leading to its accumulation in the extracellular space. Additionally, IL-1β acts directly on presynaptic terminals, increasing the probability of glutamate release. The resulting excess glutamate causes continuous neuronal depolarization, making neurons more susceptible to synchronous discharges (Sanz and Garcia-Gimeno, 2020; Kamali et al., 2021; Phan et al., 2022).

Mediation of Other Epileptogenic Factors: IL-1β stimulates glial cells to express cyclooxygenase-2 (COX-2) and mPGES-1 enzymes, which upregulate the synthesis of prostaglandin E2 (PGE2). PGE2 acts on EP receptors located on neuronal membranes, inhibiting Na^+^/K^+^-ATPase activity and thereby causing membrane depolarization and increased neuronal excitability. IL-1β also promotes the release of “danger signals” such as HMGB1 from damaged cells, further amplifying the inflammatory response via the TLR4 pathway (discussed later in the mechanism of HMGB1) (Phan et al., 2022; Radu et al., 2017;Vezzani et al., 2019).

Inducing Neuronal Changes: In animal studies, direct injection of IL-1β into the rat brain induced epileptiform discharges, whereas injection of IL-1Ra increased the convulsion threshold and reduced seizure frequency. Vezzani et al. demonstrated that sustained osmotic pumping of human recombinant IL-1Ra protein into the hippocampus, or overexpression of IL-1Ra in transgenic mice, significantly inhibited spontaneous seizures in epileptogenic states. In contrast, IL-1R1 knockout mice exhibited elevated seizure thresholds and reduced seizure severity in experimental epilepsy models. These findings strongly suggest that IL-1β/IL-1R1 signaling contributes to seizure onset, establishing IL-1β as one of the key mediators of epilepsy-associated neuroinflammation (Radu et al., 2017; Vezzani et al., 2019; Soltani Khaboushan et al., 2022; Yue et al., 2022).

### 2.2 Interleukin-6 (IL-6)

IL-6 is another pro-inflammatory cytokine closely related to epilepsy and is widely present in central and peripheral immune responses. Under normal physiological conditions, IL-6 expression in the brain is extremely low; however, IL-6 levels are significantly elevated in cerebrospinal fluid and serum of patients with epilepsy. Similar increases are observed in the hippocampus, cortex, and other brain regions of animals modelels, where levels can remain elevated for up to 24 h after status epilepticus. IL-6 is secreted predominantly by activated astrocytes and microglial cells, and its production is regulated by other cytokines, such as TNF-α, IL-1β, and IL-17, which can induce glial cells to synthesize IL-6 (Phan et al., 2022; Radu et al., 2017; Vezzani et al., 2019).

The role of IL-6 in epilepsy is more complex, but overall its pro-convulsant effect is dominant:

#### 2.2.1 IL-6 enhances excitatory synaptic transmission

Studies have shown that sustained exposure to IL-6 upregulates the expression of presynaptic glutaminase and postsynaptic AMPA receptors, promotes glutamate release and enhances excitatory synaptic pathways. At the same time, IL-6 also inhibits the formation of long-term potentiation (LTP) and reduces neurogenesis in the hippocampus, and together these effects render neural circuits more susceptible to hyperexcitability (Chen et al., 2025; Guo et al., 2022).

#### 2.2.2 IL-6 overexpression exacerbates seizure susceptibility

Selective overexpression of IL-6 in astrocytes in transgenic mice was found to be more sensitive to glutamate antagonists (e.g., kainic acid), with lower doses inducing more severe seizures. Similarly, intracerebral injection of exogenous IL-6 significantly exacerbated the intensity of seizures in models given proconvulsant GABA antagonists (e.g., zinc sulfate or bitartrate) (Guo et al., 2022; Lorigados Pedre et al., 2013; Alvim et al., 2021).

#### 2.2.3 High maternal IL-6 levels may affect the propensity of offspring to epilepsy

In one study, pro-inflammatory treatment was administered to mice during gestation, resulting in elevated IL-6 and IL-1β in the blood of the mothers, which resulted in the development of hippocampal neuronal hyperexcitability and spontaneous seizures in their offspring, suggesting that maternal inflammatory factors may influence the risk of epilepsy in the offspring via the placenta or developmental programming (Vezzani et al., 2019; Soltani Khaboushan et al., 2022; Yue et al., 2022).

However, it is important to note that IL-6’s effects on epilepsy are not all harmful. Some studies have found that mice with complete knockout of the IL-6 gene instead show increased oxidative stress, neuronal damage, and increased mortality in epilepsy models. This implies that IL-6 also possesses neuroprotective effects under certain circumstances, such as promoting tissue repair and glial scar formation after injury. Thus, the role of IL-6 is biphasic: it is predominantly proconvulsant during the acute seizure phase, whereas it may be involved in anti-injury processes during the chronic phase or in specific contexts (Hamed, 2014; Liu et al., 2023). Overall, the massive expression of IL-6 in epilepsy is undoubtedly a hallmark of central immune activation, and its main effects tend to enhance neuroexcitation and exacerbate seizures. However, given its multifaceted mechanisms. Nevertheless further studies are needed to clarify the net effect of interventions targeting the IL-6 pathway (Hermann et al., 2017; Ding et al., 2022; Zhang et al., 2024a).

### 2.3 Interleukin-17 (IL-17)

IL-17 is an epilepsy-related cytokine that has gained attention in recent years. It belongs to the pro-inflammatory mediators secreted by Th17 cells. The IL-17 family consists of isoforms ranging from IL-17A to F, with IL-17A (often abbreviated as IL-17) being the most active. Th17 cells, which play an important role in intestinal and autoimmune diseases, produce IL-17 that has been found to be significantly elevated in both epilepsy patients and experimental models. Interestingly, peripherally infiltrating Th17 cells are not the only source of IL-17 in the CNS; some glial cells and neurons also express IL-17 in epileptic pathology (Sharma et al., 2025; Soltani Khaboushan et al., 2022; Choi et al., 2021).

The effects of IL-17 on epilepsy are primarily mediated by disrupting the blood-brain barrier and triggering persistent inflammation:

#### 2.3.1 IL-17 promotes immune cell infiltration

Its receptor IL-17RA is expressed on cerebrovascular endothelial and glial cells. IL-17 binding to IL-17RA recruits the downstream Act1-TRAF6 pathway and induces the expression of chemokines (e.g., CCL2), which attracts peripheral neutrophils, monocytes, across the vasculature into the brain parenchyma. Therefore, high levels of IL-17 are often accompanied by a large number of immune cells infiltrating the brain, amplifying the inflammatory response (Choi et al., 2021; Alvim et al., 2021).

#### 2.3.2 IL-17 can inhibit GABAergic inhibition

It has been reported that IL-17 can act on the postsynaptic membrane, interfere with GABA-induced chloride influx, and attenuate inhibitory synaptic potentials. Animal brain slice experiments showed that addition of IL-17 decreased the amplitude of GABA-mediated IPSP, making neurons more likely to depolarize and generate action potentials (Alvim et al., 2021; Margetts et al., 2022).

#### 2.3.3 IL-17 and other pro-inflammatory factors promote each other

Stimulation of glial cells by IL-17 prompts them to secrete additional cytokines such as IL-6, IL-1β, TNF-α. in turn, IL-1β, upregulate IL-17 expression in glial cells. This positive feedback amplifies the local inflammatory network (Sharma et al., 2025).

#### 2.3.4 IL-17 levels correlate with epilepsy disease severity

Clinical studies have found that IL-17A concentrations in serum and cerebrospinal fluid are positively correlated with seizure frequency and the occurrence of status epilepticus in both pediatric and adult epilepsy patients. In the Kainic acid-induced epilepsy model, mice deficient in IL-17 receptors exhibited lower neuronal excitability and attenuated seizure activity.

Collectively, these findings support the view that IL-17 is an important pro-inflammatory mediator in epileptogenesis, especially in autoimmune-related epilepsy or epilepsy with intestinal immune imbalance, where the Th17/IL-17 pathway may play a critical role. Therefore, targeting and regulating Th17 cells or neutralizing IL-17 is expected to be one of the new ideas for future anti-epilepsy. However, it should be noted that excessive inhibition of IL-17 may pose an infection risk, highlighting the need for further studies to balance its potential benefits and risks (Soltani Khaboushan et al., 2022; Choi et al., 2021; Alvim et al., 2021).

### 2.4 Tumor necrosis factor-α (TNF-α)

TNF-α is a classical pro-inflammatory factor secreted by macrophages, microglia, and is equally important in epilepsy pathology. In patient brain tissue and epilepsy models, upregulation of TNF-α expression often coexists with epileptic activity and neuronal injury. TNF-α mediates its effects through its receptors TNFR1 and TNFR2. TNFR1 is widely expressed and associated with pro-inflammatory effects, whereas TNFR2 is expressed mainly on immune cells and is linked to pro-survival and regenerative functions (Kamali et al., 2021; Shan et al., 2022; Soltani Khaboushan et al., 2022). The mechanisms of action of TNF-α in epileptic conditions are complex and varied.

#### 2.4.1 Increased glutamatergic transmission

TNF-α can act on microglia and induce the upregulation of glutaminase, which promotes increased glutamate synthesis and release. In addition, TNF-α can also increase the expression and clustering of AMPA-type glutamate receptors on neuronal membranes, thus enhancing excitatory synaptic transmission (Arend et al., 2018; Hanin et al., 2023; Wang et al., 2021b).

#### 2.4.2 Impairment of GABAergic transmission

It was found that TNF-α induces endocytosis-mediated degradation of GABA_A receptors on neuronal postsynaptic membranes and reduces the number of inhibitory postsynaptic receptors. In addition, TNF-α, through its signaling, can downregulate the function of the cell adhesion molecule N-cadherin and perturb the stability of neuronal inhibitory synapses. The combined effect of both enhanced excitation and reduced inhibition makes TNF-α a potent driver of hyperexcitability within the pro-epileptic network (Arend et al., 2018; Hanin et al., 2023).

#### 2.4.3 Mediation of immune cell infiltration

TNF-α induces capillary endothelial cells in the brain to upregulate the expression of adhesion molecules (e.g., ICAM-1) and facilitates the adhesion and transmigration of peripheral T lymphocytes across the blood–brain barrier (BBB) into the brain parenchyma, thereby amplifying the local inflammatory cascade (Arend et al., 2018; Hanin et al., 2023).

#### 2.4.4 Concentration-dependent biphasic effects

Interestingly, TNF-α exerts dose-dependent biphasic effect on epilepsy. It has been experimentally reported that administration of small amounts of exogenous TNF-α in a PTZ model instead delayed seizures, while high doses of TNF-α promoted seizure onset. Further studies revealed that this may be related to differences in TNF receptor signaling. At low concentrations, TNF-α preferentially activates the high-affinity TNFR1 pathway, leading to excitotoxicity. By contrast, sustained accumulation of TNF-α can activate TNFR2, triggering anti-inflammatory and neurotrophic mechanisms that provide a degree of negative feedback and protection. Thus, during sustained seizures, TNF-α first promotes convulsions, whereas later it may induce some degree of tolerance through another pathway. This nonlinear effect is particularly important when considering anti-TNF therapy (Arend et al., 2018; Hanin et al., 2023; Wang et al., 2021b).

The antiseizure effects of TNF-α are mainly related to TNFR2 signaling, which promotes anti-inflammatory cytokines, neuronal survival, and tissue repair, thereby providing negative-feedback protection during prolonged seizures (Probert, 2015).

Overall, a large body of evidence supports a contributory role for TNF-α in seizures and epileptogenesis. Drugs that block TNF-α signaling (e.g., the TNF inhibitor infliximab.) show some seizure-reducing effects in animal models of epilepsy, but their direct clinical application remains controversial due to the wide range of biological roles and potential side effects of TNF-α.

### 2.5 High mobility group protein 1 (HMGB1) and other mediators of inflammation

HMGB1 is an intranuclear DNA-binding protein that is not normally secreted; however, in response to inflammation or cellular injury, HMGB1 can be released passively into the extracellular space or actively secreted by activated microglia, functioning as a typical Danger-Associated Molecular Patterns (DAMP) (Binabaj et al., 2019). Vezzani et al. found that HMGB1 is present in abundantly present in human and rodent epileptic focal brain tissue, and that its activation by binding to the TLR4 receptor plays a critical role in seizure generation. HMGB1-TLR4 signaling induces microglia and astrocytes to produce a cascade of inflammatory mediators, including IL-1β, TNF-α, and upregulate the production of inflammatory factors on neuronal membranes. And upregulates NMDA receptor function on neuronal membranes, increasing Ca^2+^ inward flow, leading to neuronal hyperexcitability and delayed neuronal death (Pasheva et al., 1998; Phan et al., 2022). HMGB1 has also been shown to disrupt the tight junctions of the blood-brain barrier and to induce epileptiform discharges. Moreover, experimental epilepsy models demonstrated that blockade of either HMGB1 or genetic deletion of TLR4 using neutralizing antibodies significantly reduced seizure incidence, thereby validating the pathogenic role of the HMGB1/TLR4 pathway in epilepsy (Zwilling et al., 1995; Schoenmakers and Van de Ven, 1997; Xiao et al., 2011; Ramstein et al., 1999).

Another pathway of interest is transforming growth factor-β (TGF-β) signaling. In the context of blood–brain barrier (BBB) damage, plasma albumin that enters the brain can activate TGF-β receptor I (ALK5) on astrocytes, triggering the SMAD cascade and leading to functional changes in astrocytes (Kamali et al., 2021). Specifically, under the TGF-β/ALK5 signaling pathway, the expression of Kir4.1, an inwardly rectifying potassium channel, was downregulated in the astrocyte membrane, and the polarized distribution of aquaporin 4 (AQP4) was impaired. Reduced Kir4.1 expression implies poorer potassium buffering capacity and easy accumulation of extracellular K^+^, which results in sustained excitability; and dysfunction of AQP4 affects the neurovascular unit homeostasis. Both are thought to be part of the epileptogenic effect of albumin. Thus, glial cell pathologic alterations (e.g., reactive proliferation but dysfunction of astrocytes) commonly seen in chronic epileptic foci are also associated with the involvement of inflammatory mediators (Yan et al., 2025; Hou et al., 2024).

In addition to the major inflammatory factors mentioned above, several other mediators are also implicated in epilepsy. For example, nitric oxide (NO) is a gaseous signaling molecule synthesized in response to inflammation and produced by inducible nitric oxide synthase (iNOS) (Langeh and Singh, 2021; Garg et al., 2023). After seizures, activated microglia produce large amounts of NO, which activates the cyclic guanosine monophosphate (cGMP) pathway in bystander neurons and exacerbates neuronal excitability (Kawakami et al., 2021; Kalati et al., 2022; Akyuz et al., 2021). Chemokines such as CCL2 and CXCL10 are significantly elevated in the epileptic focal brain, driving more peripheral immune cells (e.g., CCR2^+^ monocytes) into the center and amplifying the inflammatory cascade. Prostaglandin E2 (PGE2), previously mentioned, synthesized via COX-2 and mPGES-1, is also elevated during seizures and inhibits neuronal membrane pumps thereby triggering neuronal hyperexcitability. In addition, activation of the complement system has also been reported in epilepsy: C1q, C3, and other complement components accumulate in the brain of epileptic patients, and microglia express the complement receptor CR3, suggesting that complement may mediate synaptic phagocytosis and contribute to neuronal injury (Akyuz et al., 2021; Javed et al., 2022; Rahimi et al., 2022). However, the role of the complement system appears to be dual, removing necrotic debris on the one hand and damaging healthy tissue on the other. In summary, multiple inflammatory mediators collectively create a complex pro-inflammatory microenvironment within epileptic foci (as shown in Figure 1). These mediators interact and promote each other, leading to a sustained increase in neural network excitability and a heightened propensity for dysregulated activity (Javed et al., 2022; Rahimi et al., 2022; Vega Rasgado et al., 2023).

Building on the growing understanding of the role of pro-inflammatory mediators, attempts have been made in recent years to target these inflammatory pathways for intervention with a view to achieving antiepileptic effects. For example, IL-1 receptor antagonists that block IL-1β/IL-1R signaling (e.g., Anakinra) have achieved efficacy in refractory epilepsy with febrile infection-related status epilepticus (FIRES), suggesting the feasibility of this strategy. As another example, neutralizing antibodies targeting HMGB1 effectively reduced spontaneous seizures in animal models (Chao et al., 1994; Almostafa et al., 2024). Collectively, these findings underscore both the pathogenic importance and therapeutic potential of inflammatory mediators in epilepsy. Nevertheless, further clinical investigations are required to establish the safety and efficacy of such anti-inflammatory interventions in broader patient populations (Kawakami et al., 2021; Kalati et al., 2022; Akyuz et al., 2021; Javed et al., 2022).

## 3 The functional role of inflammatory cells in epilepsy

The involvement of various immune-inflammatory cells, in addition to soluble inflammatory factors, has gained increasing attention in the context of epilepsy. These cells include intrinsic immune cells within the central nervous system (CNS), such as microglia and astrocytes, as well as peripheral immune cells (e.g., monocytes/macrophages, neutrophils, T-lymphocytes) that can infiltrate the CNS following seizures. These immune cells actively shape the disease course and prognosis of epilepsy through the secretion of inflammatory mediators, cell-cell interactions, and phagocytosis (Lee et al., 2020a; Ding et al., 2022; Tröscher et al., 2023).

### 3.1 Microglia

Microglia, the resident macrophages of the central nervous system (CNS), account for approximately 5%–20% of the glial cell population in the brain (Hu et al., 2024). Originating from yolk sac hematopoietic progenitors, they perform essential myeloid functions, including phagocytic clearance, antigen presentation, and the secretion of inflammatory mediators (Tagliatti et al., 2024; Tröscher et al., 2023). In their resting state, microglia continuously survey the surrounding microenvironment through their dendritic extensions and can rapidly shift to an activated state in response to stimuli such as injury or abnormal discharges (Abbott et al., 2010; Kumar et al., 2022; Su et al., 2022). Seizure activity is a potent activator of microglia: in both human epileptic foci and animal models, they undergo a morphological transformation from a ramified, surveillant form to a hypertrophic, amoeboid “phagocytic” phenotype, accompanied by increased expression of activation markers such as Iba-1 and CD68.

Microglia activation has a dual effect on epilepsy. On one hand, excessive and sustained M1-type microglial responses exacerbate neuroinflammation and induce neuronal hyperexcitability. Studies have shown that activated microglia release large amounts of IL-1β, TNF-α, complement components, and other mediators, directly lowering seizure thresholds and contribute to neuronal damage (Kumar et al., 2022; Su et al., 2022; Wang et al., 2021a; Sanz and Garcia-Gimeno, 2020). Additionally, microglia interact with astrocytes, where inflammatory factors released by microglia stimulate the release of glutamate from surrounding astrocytes, increasing neuronal excitability and leading to excitotoxic cell death. On the one hand, microglial activation may also exert protective functions. For example, microglial proliferation during the early phase of status epilepticus facilitates clearance of cellular debris and helps limit acute injury. However, persistent microglial proliferation in the chronic phase may perpetuate neuroinflammation and promote recurrent seizures.

On the other hand, microglia also exert beneficial effects (Kiani Shabestari et al., 2022; Li et al., 2024; Massey et al., 2023). For example, they phagocytose and remove apoptotic neurons, thereby preventing the formation of aberrant neural circuits. In addition, microglia have been reported to suppress the excessive proliferation of hippocampal dentate gyrus granule cells through the TLR9–TNF pathway during the remodeling phase following epileptogenesis (Meng et al., 2023; Kiani Shabestari et al., 2022). This process reduces the generation of ectopic neurons and limits aberrant circuit formation, contributing to a reduction in seizures. Conversely, some findings suggest that inhibiting microglial activation increases the number of ectopic newborn neurons in the hippocampus after sustained epilepsy, suggesting that microglia may also contribute to pathological neurogenesis under certain conditions. These paradoxical findings may be explained by differences in epilepsy models, seizure stages, and microenvironmental factors (Zhong et al., 2022; Zhang et al., 2024b; Chen et al., 2023). The current consensus is that microglia act as “inflammatory regulators” in epilepsy. Excessive M1-type pro-inflammatory responses increasing seizure susceptibility, whereas moderate M2-type responses or clearance functions promote tissue repair and seizure suppression. Thus, microglial dysfunction—whether due to excessive inflammatory activation or impaired regulatory activity—may contribute to epileptogenesis. Modulation microglial function, for example by promoting a shift toward an anti-inflammatory phenotype or blocking specific pro-inflammatory receptors (e.g., P2X7 receptors, TLRs), has shown promise in reducing seizures in animal models. A deeper understanding of microglial roles at different stages of epilepsy will be essential for the development of relevant immunomodulatory therapies in the future (Su et al., 2022; Wang et al., 2021a; Sanz and Garcia-Gimeno, 2020).

### 3.2 Astrocytes

Astrocytes are the most abundant glial cells in the central nervous system (CNS) and play essential roles in maintaining brain homeostasis, including supporting the blood-brain barrier, regulation extracellular ion concentrations, clearing excess neurotransmitters, and providing metabolic substrates (Langeh and Singh, 2021; Su et al., 2022). In epilepsy, astrocytes undergo significantly pathological alterations. Proliferation and hypertrophy of astrocytes (i.e., gliosis, gliosis) are commonly observed in epileptic foci, representing a reactive response of brain tissue to injury or abnormal excitation (Su et al., 2022; Sanz and Garcia-Gimeno, 2020; Miller et al., 2023). Activated astrocytes can release a variety of cytokines and neurotrophic factors, and they also regulate neuronal excitability through phagocytosis and uptake of glutamate and K+. Dysregulated astrocyte function in epilepsy is primarily manifested in the following aspects:

#### 3.2.1 Impaired glutamate clearance

In models of persistent and chronic epilepsy, the function of astrocytic glutamate transporter (EAAT1/2) is downregulated, impairing timely clearance of glutamate from the synaptic cleft. The resulting extracellular accumulation leads to chronic excitotoxicity. As mentioned in the previous section on IL-1β, this mechanism is associated with inflammation-mediated suppression of EAAT expression and astrocyte injury (Sanz and Garcia-Gimeno, 2020; Miller et al., 2023; Daneman et al., 2010).

#### 3.2.2 Impaired potassium ion buffering

Under normal conditions, astrocytes take up excess synaptic K^+^ through Kir4.1 potassium channels to maintain neuronal excitatory thresholds. In epileptic foci, however, Kir4.1 expression is markedly reduced, leading to impaired local K^+^ buffering. The resulting extracellular potassium accumulation creates a hyperexcitable environment, rendering neurons more prone to depolarizing discharges (Miller et al., 2023; Daneman et al., 2010).

#### 3.2.3 Inflammatory mediator release

Astrocytes, when stimulated by epileptiform activity (e.g., repeated depolarization or IL-1β), secrete various inflammatory mediators such as IL-6, TNF-α, and CCL2. These mediators further activate the surrounding microglia and endothelial cells, promote inflammatory cell infiltration, and disrupt the BBB, thereby creating a vicious cycle (Liu et al., 2023).

#### 3.2.4 Production of seizure-promoting substances

During astrocytic proliferation, abnormal secretion of neuropeptide Y and myelin basic protein fragments has been observed, which is thought to contribute to the synchronization of epileptiform discharges (Liang et al., 2023).

#### 3.2.5 Disturbed energy metabolism

Epilepsy characterized by sustained high-frequency discharges consumes a large amount of energy, which can disrupt lactate-pyruvate metabolic coupling in astrocytes. Evidence suggests that insufficient astrocytic energy supply to neurons during status epilepticus may exacerbate neuronal metabolic stress and injury (Chen et al., 2026).

In conclusion, the role of astrocytes in epilepsy is highly complex. On one hand, they may exert protective effects through glial scar formation and metabolic support; on the other hand, their dysfunction can disrupt the extracellular environment and facilitate epileptogenesis. Importantly, astrocytes and microglia act in close synergy: microglia-derived inflammatory mediators impair astrocytic glutamate transport and potassium buffering, whereas astrocyte-derived ATP and other signals recruit and activate microglia, thereby amplifying neuroinflammatory responses.

The “glial inflammatory network” of the microglia and astrocytes the bulk of the chronic inflammatory microenvironment in epilepsy. Therefore, comprehensive interventions targeting the glial cell-neuron unit may provide a novel approach for epilepsy suppression in the future, such as restoring the excitatory/inhibitory balance by using drugs that modulate both microglial and astrocytic functions.

### 3.3 Infiltration of peripheral immune cells (macrophages, neutrophils, T lymphocytes, etc.)

Under normal physiological conditions, the blood - brain barrier (BBB) restricts the entry of peripheral immune cells into the brain parenchyma. However, in epileptic pathological states, particularly during status epilepticus or after severe trauma, the disruption of the BBB allows the migration of numerous peripheral inflammatory cells into the brain. Monocytes-macrophages and neutrophils are the most common types of infiltrating cells, and there have also been reports of T - cell infiltration (Hu et al., 2024; Ding et al., 2022; Tröscher et al., 2023).

In status epilepticus models, numerous CCR2^+^ monocytes have been observed to exit the bloodstream and infiltrate injured brain regions such as the hippocampus, where they differentiate into macrophages, with phagocytic activity and elevated expression of pro - inflammatory genes. Demonstrated that in status epilepticus models, CCR2 receptor knockout mice—lacking the receptor essential for monocyte recruitment—exhibited significantly reduced neuronal damage and cognitive deficits (Hendrix et al., 2024; Kamali et al., 2021; Phan et al., 2022). This demonstrates that brain - infiltrating monocytes/macrophages aggravate epilepsy - related brain injury. These peripherally derived macrophages share many functional similarities with central microglia and can release pro - inflammatory factors such as IL - 1β and TNF-α, exacerbating the inflammatory environment within the brain (Huppert et al., 2010; Sun et al., 2022; Vezzani et al., 2022). Moreover, they can engulf dendrites and synapses of surviving neurons, disrupting the normal architecture of neural circuits. In human epileptic foci (e.g., cortical dysplasia), a significant accumulation of CD68 - positive macrophages has been detected. These cells are likely peripherally derived macrophages that have infiltrated and, together with resident microglia, participate in the inflammatory response of the epileptic focus. Therefore, preventing abnormal infiltration of peripheral monocytes into the central nervous system or limiting their detrimental effects may provide neuroprotective and anti - epileptic benefits. For instance, studies are currently exploring the use of CCR2 antagonists or strategies to block monocyte entry into the central nervous system to alleviate post - epileptic sequelae and seizure susceptibility (Choi et al., 2021; Que et al., 2024; Spitzer et al., 2023; Chen et al., 2026).

Neutrophils are another type of immune cell that are rapidly mobilized during acute epilepsy - related inflammation. Although neutrophils are primarily active in the peripheral innate immune system, they can also infiltrate the brain during severe epileptic seizures. A research has shown that epileptic seizures can cause adhesion and accumulation of neutrophils in cerebral capillaries, leading to impaired local microcirculation perfusion. This mechanism may help explain the post-ictal hypoperfusion commonly observed in patients following seizures (Daneman et al., 2010; Abbott and Friedman, 2012; Sanz et al., 2024). Additionally, clinical studies have demonstrated that the neutrophil - to - lymphocyte ratio (NLR) in the peripheral blood of epilepsy patients is frequently elevated, reflecting the systemic inflammatory response and stress levels associated with seizures. A systematic review further confirmed that independent studies consistently reported significantly higher NLR values in epilepsy patients compared to healthy controls, with the degree of elevation correlating with seizure frequency and poor prognosis (Yue et al., 2022; Hu et al., 2023; Lagarde et al., 2022). This indirectly supports the involvement of neutrophils in epilepsy - related systemic inflammation.

Neutrophils may contribute to cerebralvascular and tissue injury through mechanisms such as the release of reactive oxygen species (ROS), secretion of proteases, and the formation of extracellular traps (NETs), thereby exacerbating epilepsy seizure susceptibility. Although the precise role of neutrophils in epilepsy has not yet been fully elucidated their status as key indicators of systemic inflammation highlights the need for further investigation (Lee et al., 2020a; Hu et al., 2024; Hermann et al., 2017). Evidence for colchicine in epilepsy is limited to case reports in familial Mediterranean fever (FMF), where seizure control improved after initiating colchicine; epilepsy-specific randomized or prospective trials are lacking (Parvez et al., 2015). Mechanistically, colchicine can reduce neutrophil recruitment/NET formation, providing a rationale for anti-inflammatory targeting (Leung et al., 2015; Vaidya et al., 2020). If proven effective, this would suggest a potential approach to modulating epilepsy - related inflammatory responses from another perspective.

T lymphocytes play a significant role in certain epileptic pathologies, such as autoimmune - related epilepsy and Rasmussen’s encephalitis. In the rare condition of Rasmussen’s encephalitis, a large number of CD8^+^ T cells can be seen infiltrating the cerebral cortex and attacking neurons. Which is thought to directly mediate epileptic seizures and progressive neurological deterioration. In more common forms of epilepsy, the role of T cells is less pronounced, but there is still evidence indicating that functional abnormalities of T - cell subsets are associated with epilepsy (Tröscher et al., 2023; Hendrix et al., 2024; Yue et al., 2022).

As mentioned earlier, IL - 17 produced by Th17 cells has a significant impact on epilepsy. A reduction in the number or function of regulatory T cells (Tregs) may weaken the suppression of abnormal inflammation and increase epilepsy susceptibility. Studies have applied IL - 2/anti - IL - 2 complexes in epileptic mouse models to expand Treg cells, resulting in reduced seizure frequency. This suggests that enhancing the immunomodulatory role of T cells may have the potential to suppress epilepsy. Overall, although peripheral T - cell infiltration into the brain is not common in most epilepsy patients, the systemic T - cell immune status (e.g., the presence of autoimmunity) can still influence the course and treatment response of epilepsy. For example, epilepsy patients with autoimmunity often respond well to immunotherapies such as steroids, IVIG, and plasma exchange. Therefore, while considering epilepsy as a disorder of local brain circuits, it is also crucial to take into account the contribution of peripheral immunity (Lai et al., 2024; Huppert et al., 2010; Geis et al., 2019). This is key to identifying certain epilepsies that may be treatable with immunotherapeutic approaches.

**FIGURE 2:**
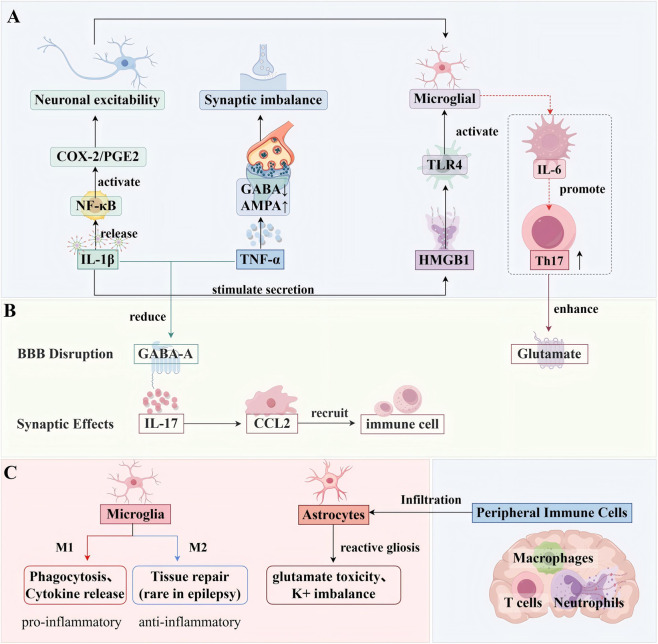
**(A)** Cytokine signaling network: Key pro-inflammatory mediators (IL-1β, TNF-α, HMGB1) form positive feedback loops:IL-1β activates NF-κB → COX-2/PGE2 pathway → neuronal hyperexcitability; TNF-α modulates synaptic AMPA/GABA receptor trafficking; HMGB1-TLR4 axis sustains microglial activation; IL-6/IL-17-Th17 amplification loop (red dashed arrows). **(B)** Neuronal dysfunction: Synaptic impairment:✓ IL-1β/TNF-α downregulate GABA-A receptors; ✓ IL-6/IL-17 enhance glutamate release; BBB disruption via IL-17→CCL2-mediated immune cell recruitment. **(C)** Cellular contributors: Microglia: Dominant M1 phenotype vs. attenuated M2 repair; Astrocytes: Reactive gliosis → glutamate/K dysregulation (↓EAAT2, ↓Kir4.1). Peripheral cells: ✓ CCR2macrophages ✓ NETs-releasing neutrophils ✓ Infiltrating Th17 cells. (By Figdraw).

In summary, various types of inflammatory cells contribute to the inflammatory microenvironment of epilepsy. Microglia and astrocytes are the core “internal players.” Their continuous activation and interaction generate a large number of pro - seizure mediators (Lee et al., 2020a; Yan et al., 2025; Abbott et al., 2010). When the BBB is compromised, “external players” such as monocytes, granulocytes, and lymphocytes enter the central nervous system, further amplifying inflammation and tissue damage (Figure 2). The combined actions of these inflammatory cells not only lower the seizure threshold and promote seizure occurrence but also contribute to the chronic progression of epilepsy and drug resistance (as sustained inflammation can alter BBB permeability and neuronal responses to drugs). This suggests that in the comprehensive management of epilepsy, in addition to traditional strategies focused on inhibiting neuronal excitability, immunomodulatory strategies targeting inflammatory cells and their products may become the next research and therapeutic focus (Chen et al., 2024b; Tang et al., 2024; Zhong et al., 2022; Wang et al., 2017).

## 4 Fundamental interactions between gut microecology and the immune system

The human gut is symbiotically populated by a diverse microbiota, including bacteria, fungi, archaea, and viruses, with a total number comparable to the cells in the human body and exceeding the number of genes in the human genome by more than a hundredfold (Diaz-Marugan et al., 2024; Mejía-Granados et al., 2021). These intestinal microorganisms establish a complex, mutually beneficial relationship with the host, thereby contributing to the regulation of host metabolism, nutrition, immunity, and other physiological processes (Zhao et al., 2024). Gut microecology influences the development and function of the host immune system through various mechanisms, with the mucosal immune system playing a key role (Olson et al., 2018). Gut-associated lymphoid tissue (GALT) is widely distributed in the intestinal submucosa, encompassing immune cell populations such as those in the Peyer’s patches, lymphoid follicles, and lamina propria. Approximately 70% of the body’s immune cells are located in GALT, highlighting the gut as one of the largest immune organs (Bemark et al., 2024; Jain et al., 2023).

Colonization of the intestinal flora during the early neonatal period is critical for immune system maturation. Germ-free mice exhibit hypoplasia of the spleen and lymph nodes and a reduced number of IgA plasma cells in the lamina propria, indicating that microbial stimulation is essential for the development of normal immune function (Vlad et al., 2024). Symbiotic bacteria interact with pattern recognition receptors (e.g., Toll-like receptors, NOD-like receptors) on intestinal immune cells via microbial-associated molecular patterns (MAMPs) (Huang et al., 2021). This interaction stimulates the mucosal barrier to produce secretory IgA, neutrophil-attracting chemokines, and antimicrobial peptides, thereby maintaining intestinal homeostasis and barrier integrity. In addition, diverse metabolic products of the intestinal microbiota, such as short-chain fatty acids (SCFAs), bile acid derivatives, and tryptophan metabolites, regulate immune cell differentiation and function (Wang et al., 2023). For instance, SCFAs, such as acetate, propionate, and butyrate, are key metabolites generated from the fermentation of dietary fiber by intestinal commensal bacteria (Yan et al., 2025). Low concentrations of SCFAs promote the expression of tight junction proteins in colonic epithelial cells by binding to receptors like GPR41/43, which enhances barrier function and stimulates anti-inflammatory cytokine production by dendritic cells and macrophages, thereby reducing inflammation. Studies have demonstrated that SCFAs, particularly butyrate acid, can induce the differentiation and proliferation of regulatory T cells (Tregs), increase anti-inflammatory cytokines like IL-10, and suppress pro-inflammatory cytokines such as IL-17 and IL-6 (Angus-Leppan et al., 2024). Maslowski et al. demonstrated that acetate supplementation reduced colonic inflammation in germ-free mice. In contrast, GPR43-deficient mice, which lack short-chain fatty acid receptors, exhibited increased colonic inflammation, elevated levels of pro-inflammatory factors (e.g., IL-17A, IL-6, IL-1β), and enhanced immune cell infiltration. These findings suggest that the intestinal commensal bacteria-SCFAs-GPR axis is a crucial mechanism in regulating intestinal immune responses (Zhai et al., 2024).

The gut microbiota, including bacteria, fungi, archaea, and viruses, plays a crucial role in regulating the immune system (Rooks and Garrett, 2016). Certain intestinal bacteria metabolize tryptophan into ligands for the aryl hydrocarbon receptor (AHR), which is expressed in dendritic cells and T helper cells (Geis et al., 2019). Activation of AHR by this ligand stimulates the secretion of pro-inflammatory cytokines, such as IL-6, from dendritic cells, and simultaneously induces Th17 cell differentiation, thereby promoting the production of inflammatory cytokines such as IL-17A (Chen et al., 2024a). Specific intestinal bacteria, such as *Streptococcus* spp. and *Lactobacillus* spp., can influence the colonization of ileal segmented filamentous bacteria (SFB), regulating the balance of Th17 cells and IL-17 levels (Lee et al., 2020b). In germ-free mice, Th17 cells are nearly absent in the spleen and intestines, but colonization with SFB restores their numbers, highlighting the crucial role of gut flora in Th17-mediated mucosal immunity. A healthy gut microbiota supports immune tolerance and reduces excessive inflammation through these mechanisms, whereas dysbiosis may cause immune dysregulation, leading to either excessive inflammation or immunodeficiency (Brooks et al., 2021).

Beyond regulating the local mucosa, gut microecology also exerts a significant influence on systemic immunity. Metabolites derived from commensal bacteria can translocate into the bloodstream and exert immunomodulatory effects on peripheral organs (Zhang et al., 2024a). For instance, butyrate—a key microbial metabolite—enters the circulation and attenuates pro-inflammatory cytokine secretion primarily through the inhibition of histone deacetylase (HDAC) activity (Wang et al., 2024). This process facilitates the polarization of peripheral monocytes toward anti-inflammatory M2-type macrophages (Liang et al., 2022). Additionally, gut microbes affect bone marrow hematopoiesis. In germ-free mice, granulocyte and monocyte production is reduced in the bone marrow, but administration of bacterial-derived components such as lipopolysaccharide (LPS) or peptidoglycan (PGN) restores myelopoiesis (Yang et al., 2023). This suggests that microbial signaling is essential for maintaining the number and function of peripheral immune cells. It is important to note that a healthy immune system maintains tolerance and homeostasis toward the gut flora. However, when intestinal barrier function is compromised or the microbiota is disrupted, microbial translocation and their products can trigger systemic immune activation and inflammation (Qi-Xiang et al., 2022).

Chronic low-grade inflammation, resulting from dysregulation of the gut-immune axis, has been linked to a variety of diseases, including autoimmune disorders, metabolic syndrome, and psychiatric conditions (Ding et al., 2022). In summary, the gut microecology and immune system engage in a delicate interplay that maintains immune homeostasis. Imbalances in this relationship can predispose the body to the development of central nervous system diseases.

## 5 How gut immune signaling affects the central nervous system

The gut–brain axis conveys peripheral physiological and pathological signals to the central nervous system, in which immune signaling acts as a critical mediator. Disruptions in intestinal immune homeostasis have been implicated in neurological disorders such as epilepsy, through effects on neuronal excitability and glial cell function mediated by humoral circulation and neural reflexes.

First, the permeability of the blood-brain barrier (BBB) plays a central role in mediating the effects of gut immune signaling (Yan et al., 2025). The BBB is composed of brain microvascular endothelial cells, basement membranes, and astrocyte end-feet, which normally prevent macromolecules and inflammatory cells from entering the brain parenchyma, thereby maintaining central nervous system homeostasis (Abbott et al., 2010; Salvador et al., 2021). However, disruptions in gut microbiota can impair the integrity of the BBB. Studies show that germ-free or antibiotic-treated mice exhibit significantly reduced expression of tight junction proteins (e.g., Occludin, Claudin) in brain microvascular endothelial cells, leading to increased BBB permeability (Zeng et al., 2024; Simpson, 2023; Daneman et al., 2010; Wolka et al., 2003). A bone marrow transplantation study further revealed that mice with antibiotic-cleared microbiota are more susceptible to peripheral monocyte infiltration into the brain after transplantation. These findings suggest that gut microbiota are essential for maintaining BBB integrity (Wolka et al., 2003; Abbott and Friedman, 2012; Rapoport, 1996).

Mechanistically, this may be linked to microbial metabolites, such as short-chain fatty acids, which promote tight junction protein expression. Additionally, certain cytokines produced by commensal bacteria can stimulate the gut. For instance, butyrate upregulates tight junction protein expression in brain endothelial cells, reducing BBB permeability and thereby limiting the invasion of brain tissue by pathogens and inflammatory mediators (Ballabh et al., 2004; Friedman and Kaufer, 2015; Wang et al., 2017). Conversely, during intestinal inflammation, high levels of pro-inflammatory cytokines (e.g., IL-1β, TNF-α, IL-6) and endotoxins (e.g., LPS) can enter the bloodstream, targeting the endothelial cells of cerebral microvasculature. This induces the secretion of matrix metalloproteinases, which cleave tight junctions and compromise BBB integrity. Increased BBB permeability facilitates the infiltration of peripheral immune cells and plasma proteins into the brain.

Classic experiments by Seiffert et al. demonstrated that repeated injections of serum albumin into the rat cortex, mimicking BBB leakage, can generate epileptic foci at the injection site and induce spontaneous seizures (Davis et al., 2019). Further research revealed that albumin entering the brain binds to TGF-β receptors on astrocytes, initiating a cascade that prompts astrocytes to secrete large amounts of pro-inflammatory molecules, such as IL-6 and TNF-α. These inflammatory factors significantly increase neuronal excitability and lower the threshold for seizures (Campisi et al., 2021). Thus, intestinal-derived pro-inflammatory mediators profoundly influence epileptogenesis by disrupting the BBB and triggering central nervous system inflammation, forming a cycle of “intestinal inflammation - BBB disruption - central inflammation.”

Additionally, intestinal immune signals can directly affect brain neuronal activity via vagal reflexes. Infections or inflammation in the intestinal mucosa can activate sensory fibers of the enteric nervous system and vagus nerve, transmitting signals to structures such as the nucleus tractus solitarius in the medulla oblongata. These signals subsequently alter the excitability of neural networks in the brain (Simpson, 2023; Daneman et al., 2010; Wolka et al., 2003). In animal models of intestinal inflammation, altered vagal firing patterns and elevated levels of inflammatory mediators in the brain have been observed, suggesting that gut inflammation can activate central inflammatory responses and modify neural excitability through neural pathways (Zeng et al., 2024). However, research on the role of the vagus nerve in the gut-brain axis in epilepsy remains limited. Nevertheless, evidence from vagus nerve stimulation therapy in refractory epilepsy suggests that vagal modulation can influence brain excitability and potentially modulate inflammatory states (Yan et al., 2025; Abbott et al., 2010; Salvador et al., 2021).

In addition, the regulation of the central immune environment by metabolites from the intestinal microbiota is an essential factor that should not be overlooked. Beyond their peripheral effects, previously mentioned short-chain fatty acids can cross the blood-brain barrier (BBB) via monocarboxylic acid transporter proteins to enter the brain (Lee et al., 2020a). In the brain, butyric acid binds to the GPR109A receptor on microglia, inhibiting their pro-inflammatory activation and enhancing neuronal function (Shan et al., 2022; Lehner et al., 2011). Animal studies have shown that butyric acid administration reduces microglial inflammation and improves cognitive function in mice chronically exposed to alcohol. Alternatively, propionic acid promotes the nuclear translocation of the antioxidant transcription factor Nrf2 in glial cells, reducing reactive oxygen species (ROS) levels and protecting the BBB from inflammation and oxidative stress (Yang et al., 2024; de Mello et al., 2018; He et al., 2022). Moreover, indole derivatives produced from tryptophan metabolism by intestinal microbiota act as AHR agonists (He et al., 2022; Du et al., 2022; Wei et al., 2024). These derivatives cross the BBB and bind to AHR receptors on mesangial microglia and astrocytes. Rothhammer et al. demonstrated that activation of microglia through the AHR pathway upregulates pro-inflammatory molecules, such as tumor growth factor-α (TGF-α) and vascular endothelial growth factor B (VEGF-B). These molecules modulate the functional phenotype of astrocytes by interacting with surface receptors, where VEGF-B promotes astrocyte pathogenicity and inflammation, while TGF-α exerts an opposing effect (Abbasloo et al., 2023; Chen et al., 2024b; Tang et al., 2024). This suggests a crosstalk axis between gut-derived metabolites, microglia, and astrocytes. An imbalance in this regulation, particularly when specific intestinal bacteria are absent, may lead to an overproduction of pro-inflammatory astrocytes and an increased susceptibility to epilepsy (Tang et al., 2024). Notably, significantly elevated levels of IL-17 in peripheral blood and cerebrospinal fluid are commonly observed in patients with epilepsy and correlate positively with seizure frequency and severity (Sharma et al., 2025). The fact that IL-17 is predominantly derived from gut-bacteria-driven Th17 cells further supports the impact of gut immunity on central nervous system disorders: dysbiosis of the gut microbiota induces excessive Th17/IL-17 axis activation, which enters the brain via the bloodstream, promoting neuronal hyperexcitability and seizures (Soltani Khaboushan et al., 2022; Sharma et al., 2025; Choi et al., 2021; Margetts et al., 2022; Huppert et al., 2010). In summary, gut-immune signaling influences the brain through multiple pathways: on the humoral pathway, intestinal inflammatory products disrupt the BBB and act directly on brain cells; on the neural pathway, enteric neuro-vagal reflexes alter central nervous system activity; and on the metabolic pathway, bacterial products modulate glial and neuronal function across the BBB. Together, these pathways form the biological basis of intestinal influence on epileptogenesis. With a deeper understanding of the gut-brain axis, it is evident that epilepsy is not an isolated pathological process confined to the brain, but rather a syndrome involving multiple systems throughout the body, in which the gut-immune system, in particular, plays a crucial role (Figure 1).

## 6 Clinical research and therapeutic strategies

In recent years, clinical research on epilepsy patients has increasingly confirmed the importance of neuroimmunological mechanisms in the disease. These studies, which serve as the basis for translating basic findings into clinical applications, can be categorized into several key areas: detecting inflammatory biomarkers in patients’ biological samples, observing the clinical characteristics of epilepsy coexisting with immune-related diseases, and conducting experimental treatments targeting inflammatory pathways (Lee et al., 2020a; Arredondo et al., 2024).

### 6.1 Inflammatory biomarkers in epilepsy patients

Numerous clinical studies have reported significantly elevated levels of multiple inflammatory factors in the peripheral blood and cerebrospinal fluid (CSF) of patients with active epilepsy. For instance, as early as 1998, Peltola et al. found that IL-6 levels in patients’ CSF rapidly increased shortly after a seizure, positively correlated with seizure duration. Subsequently, Mao et al. analyzed the cytokine profiles in the peripheral blood and CSF of epilepsy patients and found that levels of inflammatory factors such as IL-1β, IL-6, IFN-γ, and IL-17A were significantly higher than in healthy controls. Among these, IL-17A levels showed a positive correlation with annual seizure frequency and severity. These findings established that epilepsy patients exhibit systemic and central inflammatory activation (Kanemura, 2024; Vinti et al., 2021; Chang et al., 2021).

Additional inflammatory markers have since been proposed as potential biomarkers for epilepsy. For example, the neutrophil-to-lymphocyte ratio (NLR), mentioned earlier, has consistently been reported to be elevated across multiple independent cohorts in epilepsy patients and shows a stronger association with refractory epilepsy. Non-specific inflammatory indicators such as C-reactive protein (CRP) and erythrocyte sedimentation rate (ESR) may also be elevated in some epilepsy patients, particularly those with systemic infections or autoimmune comorbidities. Additionally, the positivity rate of anti-neuronal autoantibodies (e.g., anti-glutamate receptor antibodies, GAD antibodies, etc.) is higher in epilepsy patients of unknown etiology compared to the general population, supporting the notion that a subset of epilepsies may have an autoimmune-inflammatory basis.

Although inflammatory markers do not possess sufficient specificity to function as stand-alone diagnostic tools for epilepsy, they offer additional insights into patients’ pathophysiological states. In the future, integrating multiple markers into a composite ‘epilepsy inflammation score could help guide personalized immunotherapeutic strategies (Wan et al., 2021; Khedpande and Barve, 2025; Auvin et al., 2025; Tanaka et al., 2024).

### 6.2 Autoimmune and inflammation-related epilepsies

A subset of epilepsies is closely linked to immune-mediated encephalitis. A typical example is autoimmune limbic encephalitis, which frequently presents as anti-NMDA receptor encephalitis or anti-LGI1 encephalitis. In many cases, epileptic seizures constitute the initial symptom, followed by cognitive decline and psychiatric manifestations. These cases share several common features: detection of autoantibodies against neuronal antigens in cerebrospinal fluid (CSF) and serum, increased inflammatory cells and protein in CSF, and brain imaging showing encephalitis changes. Immunosuppressive therapies (e.g., glucocorticoids, IVIG, rituximab, etc.) usually significantly alleviate seizures and symptoms, indicating that autoimmune-mediated inflammation is a key pathogenic factor (Tanaka et al., 2024; Riva et al., 2024a; Ravizza et al., 2024).

Beyond definitive cases of autoimmune encephalitis, some patients with refractory epilepsy may have occult immune mechanisms. For example, Rasmussen encephalitis, which typically onsets in childhood, is characterized by progressive unilateral cerebral hemisphere atrophy and intractable seizures. Pathologically, it is marked by T lymphocyte-mediated inflammation and neuronal injury. Such patients sometimes respond to high-dose glucocorticoids or immunosuppressive therapy, but often require surgical resection of the affected area for a definitive treatment. These cases highlight the importance of targeting immune-mediated inflammation in certain epilepsies (Tang et al., 2024; Zhong et al., 2022; Vezzani et al., 2022).

Some scholars have proposed the concept of “autoimmune-related epilepsy syndromes” to describe epilepsy patients who have autoimmune diseases (e.g., systemic lupus erythematosus, gluten intolerance, etc.) or neuro-autoantibodies. For these patients, adding immunomodulatory therapy to standard anti-epileptic drugs may improve prognosis (Symonds et al., 2021; Scott, 2021; Tang et al., 2024). Indeed, some refractory epilepsy patients who failed to respond to conventional anti-epileptic medications have shown success with immunotherapy. For example, in adult refractory epilepsy patients with autoimmune thyroiditis, the addition of immunosuppressants significantly reduced seizure frequency. Although such evidence is currently mostly anecdotal, it suggests the need for immunological evaluation in refractory epilepsy patients to identify potential treatable immune triggers (Tanaka et al., 2024; Riva et al., 2024a; Ravizza et al., 2024).

### 6.3 Exploring anti-inflammatory therapies for epilepsy

Based on the aforementioned mechanisms, clinical exploration of various anti-inflammatory and immunomodulatory therapies for refractory epilepsy has gained momentum in recent years. One notable attempt is the use of the IL-1 receptor antagonist Anakinra in children with FIRES (febrile infection-related epilepsy syndrome). FIRES is a catastrophic pediatric epilepsy syndrome characterized by intractable epileptic status epilepticus following a fever, often leaving severe sequelae. Several recent clinical reports indicate that adding intravenous Anakinra to conventional sedation and anti-epileptic regimens can significantly reduce seizure frequency and improve consciousness in children. These observations support the critical role of IL-1-mediated inflammatory storms in FIRES and suggest that blocking IL-1 signaling may suppress abnormal discharges (Ihezie et al., 2021; Chen et al., 2024c; Symonds et al., 2021).

Similarly, the IL-6 neutralizing antibody Tocilizumab has been administered in the chronic phase treatment of children with FIRES. A study involving several chronic-phase FIRES patients reported that Tocilizumab reduced seizure frequency and severity in some cases (Aledo-Serrano et al., 2022; Donnelly et al., 2021; Wadayama et al., 2021; Girardin et al., 2023). In post-encephalitic epilepsy or autoimmune-related epilepsy, immunotherapies such as glucocorticoids and intravenous immunoglobulin (IVIG) have also been effective in controlling seizures and, in some cases, achieving remission. For example, in patients with anti-LGI1 encephalitis, long-term low-dose corticosteroid maintenance following acute-phase seizures has been reported to prevent seizure recurrence.

In addition to systemic medications, local anti-inflammatory strategies are also under investigation (Nangia et al., 2024; Chen et al., 2026; Gotra et al., 2021). For instance, Dutch researchers injected inflammation-suppressing neuropeptides delivered via lentiviral vectors into the hippocampus of mice and found that it reduced seizure frequency. Given the potential side effects of systemic anti-inflammatory drug use, such as increased infection and tumor risks, local drug delivery or controlled-release carrier methods may represent one of the future research directions (Wirrell et al., 2022; Lorigados Pedre et al., 2013; Mula et al., 2022).

### 6.4 Gut microbiota-targeted therapies

Considering the role of the gut-immune axis in epilepsy, gut microbiota-targeted therapies are gaining increasing attention. The most well-known approach is the ketogenic diet (KD), a high-fat, low-carbohydrate dietary intervention with significant efficacy in refractory epilepsy, particularly in children. The exact mechanism of KD has long been unclear, but it is now widely accepted that KD may exert its effects by reshaping gut microbiota and metabolites (Ding et al., 2022; Yan et al., 2025; Zhang et al., 2024a).

Research by Olson et al. revealed that KD treatment increases the proportion of two specific gut bacterial taxa in mice: Akkermansia muciniphila (phylum Verrucomicrobia) and Parabacteroides (phylum Bacteroidetes). When these two bacteria coexist, they can enhance the gut’s capacity to produce gamma-aminobutyric acid (GABA), thereby reducing seizure frequency. Importantly, the anti-seizure effect of KD was abolished when to deplete gut microbiota or when KD was administered to germ-free mice, the anti-seizure effects of KD disappeared, highlighting the indispensability of gut microbiota (Li et al., 2025; Qi-Xiang et al., 2022; Chen et al., 2024a).

Beyond KD, probiotics and prebiotics (substrates that promote the growth of beneficial bacteria) are being investigated as adjuvant therapies for epilepsy. A small-scale randomized controlled trial administered a probiotic formulation containing *Lactobacillus acidophilus*, *Bifidobacterium*, and other beneficial bacteria to drug-resistant epilepsy patients. Approximately 28% of patients in the treatment group experienced a >50% reduction in seizure frequency, whereas no significant improvement was observed in the control group. Although the sample size was limited, these finding suggests that gut microbiota modulation may lower seizure susceptibility in some patients (Riva et al., 2024b; Mejía-Granados et al., 2021; Mazarati, 2024).

Additionally, several case reports have documented the potential benefit of fecal microbiota transplantation (FMT) in patients with epilepsy and coexisting conditions. For example, a case report described a patient with refractory epilepsy whose seizure frequency dramatically decreased and remained reduced for over 6 months following FMT for *Clostridium difficile* infection. While it is unclear whether this outcome was coincidental or causally related, it has sparked interest in FMT as a potential epilepsy therapy (Chen et al., 2024a; Diaz-Marugan et al., 2024; Riva et al., 2024b).

Overall, gut microbiota–modulating therapies for epilepsy are still in the exploratory phase, with their mechanisms of action, target populations, and long-term safety requiring further investigation. However, as a manipulable entity, gut microbiota has the potential to become an emerging field in future epilepsy management (Wang et al., 2024; Liang et al., 2022).

### 6.5 Other potential anti-inflammatory targeted strategies

In addition to the aforementioned therapies directly targeting cytokines or gut microbiota, some commonly used medications have also demonstrated dual anti-inflammatory and anti-epileptic effects (Hermann et al., 2017; Arredondo et al., 2024). For example, metformin, an antidiabetic drug, has been found to activate the adenosine pathway and attenuate microglial inflammation. In animal epilepsy models, it reduced seizure frequency. Statins, lipid-lowering drugs with anti-inflammatory and endothelial function improvement properties, have been reported to alleviate post-epileptic cognitive impairments in when administered after status epilepticus. These “drug repurposing” findings highlight promising avenues for anti-inflammatory epilepsy treatment. However, these strategies require rigorous clinical trial validation (Gotra et al., 2021).

Finally, for some definitive inflammation-related epilepsies, surgical treatment remains a crucial option. For instance, in advanced Rasmussen encephalitis, hemispherectomy is often necessary to radically cure seizures due to irreversible inflammatory damage. Early identification and intervention in inflammatory mechanisms are thus critical to avoiding surgical outcomes.

## 7 Conclusion and future directions

Neuroimmune interactions within the gut-brain axis are critical in epileptogenesis, with mechanisms spanning gut dysbiosis, systemic inflammation, and central neuroinflammation. Key pro-inflammatory factors (e.g., IL-1β, TNF-α, HMGB1) and immune cells (microglia, astrocytes, peripheral macrophages) combine to cause neuronal hyperexcitability and blood-brain barrier (BBB) disruption in a self-perpetuating cycle of ‘intestinal inflammation–BBB damage–central inflammation. Therapeutic strategies targeting these pathways (e.g., IL-1R antagonist Anakinra, HMGB1 inhibitors, ketogenic diets to regulate bacterial flora, probiotics) have shown potential in preclinical and selected clinical studies (e.g., FIRES syndrome). In summary, clinical research has provided substantial evidence supporting the significant role of inflammatory factors and cells in epilepsy, with preliminary immunomodulatory treatments showing promise. However, most immunotherapies are currently applied only in specific subgroups or critical cases. Demonstrating their safety and efficacy in a broader epilepsy population remains a significant challenge. Further in-depth mechanistic studies should be conducted to elucidate potential therapeutic targets for epilepsy prevention and treatment.
